# Serum‐Free Japanese Encephalitis Virus Production in a Single‐Use Fixed‐Bed Bioreactor

**DOI:** 10.1002/elsc.70052

**Published:** 2025-10-22

**Authors:** Marco Kress, Robert Schlegl, Alois Jungbauer

**Affiliations:** ^1^ Austrian Centre of Industrial Biotechnology Vienna Austria; ^2^ Department of Biotechnology BOKU University Vienna Austria

**Keywords:** iCELLis, Japanese encephalitis virus, OptiPRO SFM, roller bottles, Vero

## Abstract

Fixed‐bed bioreactors for anchorage‐dependent cells are an obvious choice for development because of their large‐scale capabilities, allowing manufacturing with reduced cost and footprint. In this study, a serum‐free production process for Japanese encephalitis virus (JEV) in a single‐use fixed‐bed bioreactor was developed and compared to conventional roller bottle production as a productivity benchmark. After optimization of serum‐free cell culture conditions, an initial media screening in roller bottles showed a strong impact of growth and production media on virus yields. Selected optimized medium combinations were assessed in roller bottles and the fixed‐bed bioreactor. Both systems proved to be excellent production systems for JEV, but media choice was key to achieve the highest titers. In particular, DMEM with its enriched glucose content beneficially affected viral yields, enabling potential large‐scale manufacturing using the fixed‐bed reactor with serum‐containing or serum‐free media.

*Practical application:* Data presented in this work show feasible ways of serum‐free virus production with Vero cells, a common cell substrate in vaccine development. The fixed‐bed bioreactor process described here could facilitate manufacturing activities to reduce cost and footprint while simultaneously achieving higher process control compared to conventional manufacturing systems like roller bottles. With a much better upscale potential (up to 500 m^2^) the fixed‐bed bioreactor showed comparable or better yields to roller bottles depending on media used, even with serum‐free media. This research article further emphasizes the need to optimize cell culture media or media combinations for each virus individually to achieve the highest titers. As shown, performing a simple media screening experiment to optimize yields early in process development could lead to better productivity, with a high business impact in later development stages.

**Abbreviations**
CPE: cytopathic effect
JEV: Japanese encephalitis virus
MOI: multiplicity of infection
SE‐HPLC: size‐exclusion HPLC
SFM: serum‐free media

## 1 Introduction

Modern vaccine production must meet many standards to ensure delivery of safe and effective products. State‐of‐the‐art manufacturing with reduced cost and footprint is required for ensuring improved vaccine access in lower‐income countries. Fixed‐bed bioreactors offer an obvious option with considerable large‐scale capabilities. A lack in process understanding, however, hampers their broader use and development data and manufacturing experience are still needed. The need can be seen, for example, in increasing efforts in process‐analytical technologies and quality‐by‐design studies to understand these processes [1]. In addition, new manufacturing technologies are clearly needed to lower the cost of goods while also increasing productivity and quality. For a cell culture‐based production system using anchorage‐dependent cells, fixed‐bed bioreactors yield the highest cell densities in a compressed and static fixed‐bed maintained by perfusion or media recirculation. In this way, the floor space of the manufacturing plant can be reduced. A growth area that is several hundred square meters can be maintained with a very small footprint, as has been seen with adeno‐associated virus or lentivirus production for gene‐therapy usage in an iCELLis 500 bioreactor [2, 3]. Manufactured by Cytiva, this system is a commercially available single‐use fixed‐bed bioreactor for the cultivation of adherent cells. The static fixed‐bed is filled with macrocarrier strips (polyester microfibers), pre‐packed as a single‐use unit in different sizes ranging from 0.5 to 4 m^2^ growth area for the development version, iCELLis Nano, or 66–500 m^2^ growth area for the large‐scale system (iCELLis 500+), equivalent up to 5900 roller bottles. Due to its unique design, the cell culture medium flows upwards through the fixed‐bed and falls as a thin film down the outer wall, resulting in high oxygenation rates while keeping shear stress levels low. The compact 3D‐environment of the macrocarriers allows for significant reduction in seeding densities, leading to simpler seed train designs and further footprint reduction compared to stirred‐tank bioreactors, for which seeding bioreactors may be necessary for large‐scale production [4, 5].

Many licensed vaccines are still made using old‐fashioned production process designs, such as chicken eggs or roller bottles as the manufacturing platform. Japanese encephalitis virus (JEV), which can be produced in roller bottles on Vero cells, is a leading cause of viral encephalitis in Asia, with more than 10,000 deaths per year [6, 7]. The roller bottle production system has limited scalability and many handling steps, requiring manual intervention or robots and entailing a substantial upfront investment [8, 9]. One solution is to render such a process in a completely new format using single‐use bioreactors like the iCELLis system.

State‐of‐the‐art manufacturing of viral vaccines should use serum‐free media (SFM), as is strongly recommended by health agencies to avoid the risk of adventitious agents. Thus, serum requires extensive and cost‐intensive testing, and inherent lot‐to‐lot variations can affect production [10, 11]. Nonetheless, serum is an important source of nutrients, hormones, and growth factors and promotes cell attachment and spreading on the surface, along with protecting cells from shear‐induced damage [12]. More sensitive cell growth is usually associated with SFM, requiring additional optimization efforts. Examples of successfully implemented Vero cell processes using SFM include Baxter BioScience's influenza vaccine production, which was reported to manufacture up to a 6000 L scale, or the licensure of Ervebo against Ebola virus disease [5, 13]. Most SFMs are animal‐free and have a low protein concentration, but a full chemical characterization is elusive because various hydrolysates derived from plant sources, such as rice or soy, as serum substitute yield a non‐defined mixture [14, 15]. Complete chemically defined formulations for Vero cells are mostly still research‐based and high cost and cannot supply commercial manufacturing.

A modern single‐use production system, complete animal‐free production, and efficient large‐scale manufacturing without challenging seeding strategies are all requirements for a contemporary vaccine production process. Our goal was to test SFM in a scalable bioreactor to identify an ideal production system with suitable media combinations for individual virus production. For this purpose, we developed a process that relies on modern single‐use bioreactor technology with an iCELLis system, along with SFM, and compared it to conventional roller bottle production for optimal overall productivity for JEV manufacturing.

## 2 Materials and Methods

### 2.1 Cell Line Propagation and Virus Seed

Vero cells (#CCL‐81, ATCC) were used in all experiments. Cultivation of serum‐enriched cells was done in MEM or DMEM‐HG with 10 % FBS (Biochrom AG) (5 % iCELLis Nano). Serum‐free production was conducted in VP‐SFM or OptiPRO SFM, with serum‐free cell banks produced beforehand by direct adaptation over 10 passages. All media were supplied by Gibco (Thermo Fisher Scientific) as were L‐Glutamine, PBS, and TrypLE.

Cells cultivated with FBS were washed twice with PBS and detached by TrypLE for 15 min at 37°C. Standard serum‐free cell passaging consisted of a single PBS wash followed by Accutase (Capricorn Scientific) addition and its removal after 1‐2 min before continuing incubation at room temperature for 12–15 min. All cell counts were performed using a NucleoCounter NC‐200 (ChemoMetec).

As viral seed, we used one of Valneva's JEV seed banks based on the live‐attenuated JEV SA14‐14‐2 strain, from which several JEV vaccines have originated [6].

### 2.2 Production Systems

As standard plasticware, Corning CellBIND‐treated T‐150 flasks and corresponding roller bottles (850 cm^2^) were used. Incubation was performed at 5 % CO_2_ and 37°C. Roller bottles were incubated in an Incudrive 90 incubator (Schuett‐Biotec, Germany) at 0.5 rpm (0.3 rpm SFM cells).

For cultivation, an iCELLis Nano (Cytiva) fixed‐bed bioreactor equipped with a 1.07 m^2^ low compaction single‐use unit was used in combination with an Applikon my‐Control unit (Software mE.2.9) for cultivation. Preparation was performed as described by the manufacturer, including bioreactor installment, steam sterilization, media addition (850 mL working volume), and carrier conditioning. Afterwards, 10^4^ cells/cm^2^ were seeded, provided from a roller bottle as the final container. To enhance cell attachment, an increased stirrer speed was applied for 3 h. Process parameters were set to 37°C, pH 7.40, and 50 % dissolved oxygen. On Day 2 or 3 after seeding, a recirculation bottle was connected to maintain a volume‐to‐surface ratio of 5 L/m^2^ until the infection timepoint. During daily sampling, the pH, metabolites (BioProfile pHOx and BP 100+, Nova Biomedical), and cell counts were determined. For cell counts, a minimum of two macrocarrier strips were removed from the fixed‐bed and treated with reagents for cell release and subsequent stabilization, as described by the NucleoCounter NC‐200 manufacturer. Before viral infection, media recirculation was discontinued, and two 800 mL PBS washes were performed. Production media was added (working volume 850 mL), and infection was conducted at 35°C by direct pipetting of the viral suspension (multiplicity of infection [MOI] 0.01) into the fixed bed. Harvests with media exchanges were executed on Days 3, 5, 7, and 9 post‐infection.

### 2.3 Media Optimization

As media, MEM, DMEM, OptiPRO SFM, and VP‐SFM were combined and tested in 16 groups to assess viral productivity. MEM and DMEM were supplemented with 10 % FBS (growth phase only) and 2 or 4 mM L‐Glutamine, OptiPRO SFM, and VP‐SFM with 4 mM L‐Glutamine alone. Four individual seed trains were propagated in T‐flasks for three passages until cultivation in roller bottles. At the time of infection, cell counts were determined, spent media removed, two PBS washes conducted, new media added, and all roller bottles (two per group) infected with JEV (MOI 0.01). Production temperature was lowered to 35°C, and multiple harvests with media refill were performed at Days 3, 5, and 7 until the end of fermentation at Day 9 post‐infection.

### 2.4 Virus Quantification by SE‐HPLC

As the analytical method, we used size‐exclusion high‐performance liquid chromatography (SE‐HPLC) on a Vanquish HPLC (Thermo Fisher Scientific) with a Superose 6 Increase 10/300 GL (Cytiva) column to determine the amount of JEV particles. Wavelength peaks were recorded at 214 nm absorbance using Chromeleon 7.2 software (Thermo Fisher Scientific). Because JEV is an enveloped flavivirus with 50 nm in diameter, it can be separated from smaller particles by this method [7, 16]. As the mobile phase, PBS with 250 mM NaCl (VWR) pH 7.4 was prepared, running at 1 mL/min with a 100‐µL sample injection volume. To reduce nonspecific adsorption effects, 50 µg/mL bovine serum albumin (Thermo Fisher Scientific) was added to each sample.

For this analysis, the void volume purity is decisive because anything with a size similar to that of the virus particle will be automatically co‐detected. To ensure purity, all samples were precipitated with 2 mg/mL protamine sulfate (Yuki Gosei Kogyo, Japan) and centrifuged at 3000 × *g* for 15 min to remove host cell DNA, larger proteins, and excess cell debris beforehand.

Supplementary supernatant analysis by multi‐angle laser light scattering and dynamic light scattering confirmed high purity (> 90%) in size distribution, indicating that this procedure was sufficient to achieve appropriate purity levels for subsequent SE‐HPLC analysis (data not shown).

## 3 Results

Establishment of serum‐free cell culture using OptiPRO SFM or VP‐SFM, including enzyme and detachment optimization, was performed before the media optimization in roller bottles. The aim was to find high‐performing media combinations and to confirm them in the iCELLis bioreactor. Finally, a productivity comparison of JEV manufacturing between the roller bottle and iCELLis systems was made.

### 3.1 Enzyme and Detachment Optimization

Initially, adjustments of culture conditions and the detachment procedure were necessary for cells cultivated in SFM because of their sensitive nature, which can result in unstable cell growth. A weaker cell adhesion ability was noted for cells cultured in serum‐free media, which was more pronounced in roller bottles than in T‐flasks. This was solved by decreasing roller bottle's rotation speed along with optimization of the detachment procedure. Thus, TrypLE and Accutase were evaluated as enzymes in addition to the application of three different detachment procedures. The first procedure was similar to the serum‐enriched method. Incubation was stopped by adding media (without serum for SFM). The second procedure consisted of enzyme solution removal by aspiration after 1‐2 min. As a modification of the first procedure, the third procedure consisted of an additional centrifugation step after incubation to completely remove the detachment solution instead of diluting it.

**FIGURE 1:**
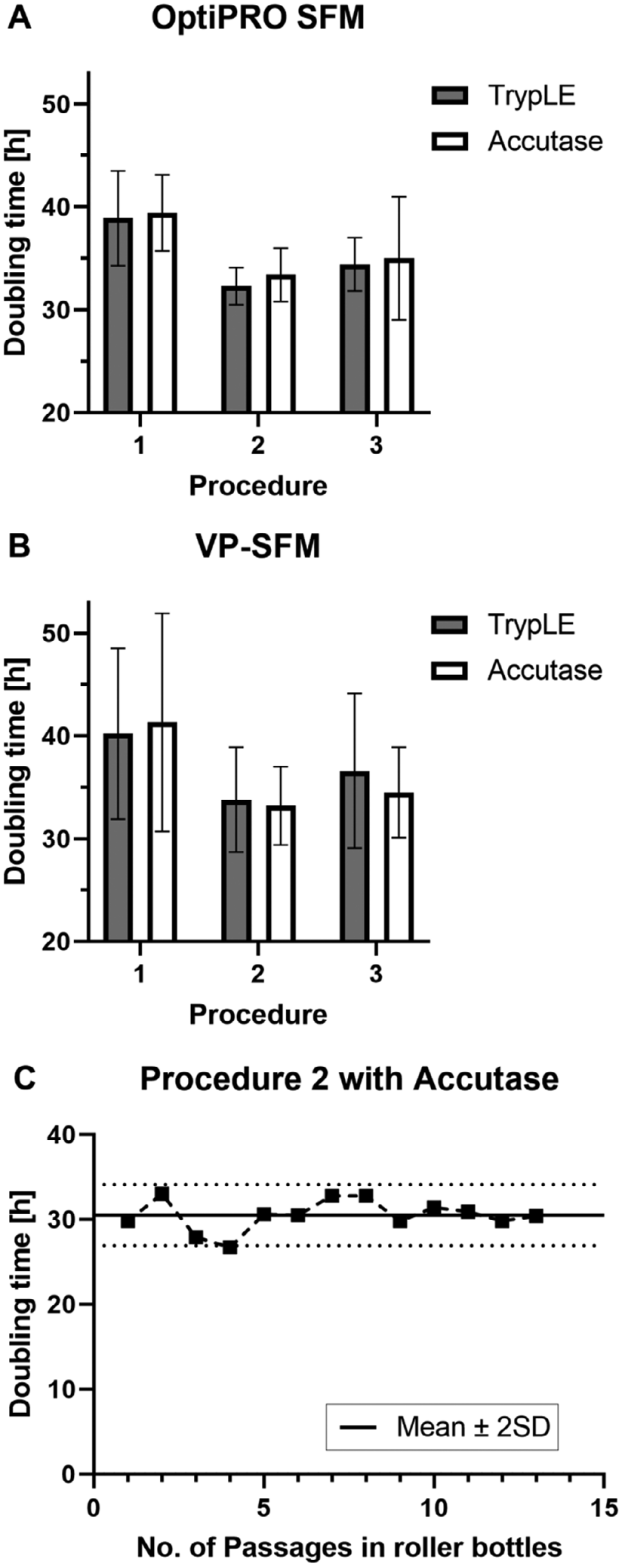
Effects on doubling times of TrypLE (grey bar) and Accutase (white bar) in three cell detachment procedures (1–3) using OptiPRO SFM (A) or VP‐SFM (B) over nine passages in T‐flasks. Procedures: (1) incubation stopped with the used SFM; (2) enzyme removed after 1–2 min incubation; and (3) enzyme removal by centrifugation. One‐fold standard deviation (SD) is shown for (A) and (B). (C) Doubling times of Procedure 2 using Accutase in roller bottles (VP‐SFM) over 13 passages (black squares, dashed line), highlighting the mean value ±2 SD (black solid line and black dotted lines).

Evaluation of the three detachment procedures and two enzyme solutions was performed based on how they affected calculated doubling times for cultivation in OptiPRO SFM or VP‐SFM. Results from T‐flasks were compared over multiple passages (nine in total), as shown in Figure 1.

TrypLE and Accutase both can be used for Vero cell detachment in SFM, maintaining low and stable doubling times in subsequent passages if the correct detachment procedure is used. For Procedure 1, the detachment solution was kept and diluted with used media after incubation, which resulted in doubling times of 38.9 h (OptiPRO SFM) and 41.3 h (VP‐SFM), but with a high standard deviation (SD) for both. Removal of the enzymes led to lower doubling times, with removal by aspiration after 12 min (Procedure 2) or centrifugation after incubation (Procedure 3). Procedure 3 yielded doubling times around 35 h, with a higher SD compared with Procedure 2. Doubling times were more stable around 33 h for both media. Moreover, we noted higher aggregation levels (clumped cells) with TrypLE compared to Accutase, which required fewer resuspension steps to break visible cell clumps after detachment.

Finally, given the consistent results and lowest SD for both media tested, we chose Procedure 2 in combination with Accutase as the standard detachment method. This choice was confirmed by additional roller bottle data, as shown in Figure 1C. These data indicated a mean doubling time of 30.5 h in the range of 2× SD over 13 passages in VP‐SFM, confirming robust doubling times with this optimized detachment method.

### 3.2 Roller Bottle Media Screening

The best growth and production media combination for JEV in roller bottles was developed to obtain the highest productivity before rendering the process in a completely new format, the iCELLis Nano. Four different seed trains were started, resulting in slightly faster doubling times for FBS‐cultured cells, with 29 h on average for MEM (29.2 h) and DMEM (28.7 h) compared to 33 h on average for OptiPRO SFM (33.8 h) and VP‐SFM (33.0 h).

**FIGURE 2:**
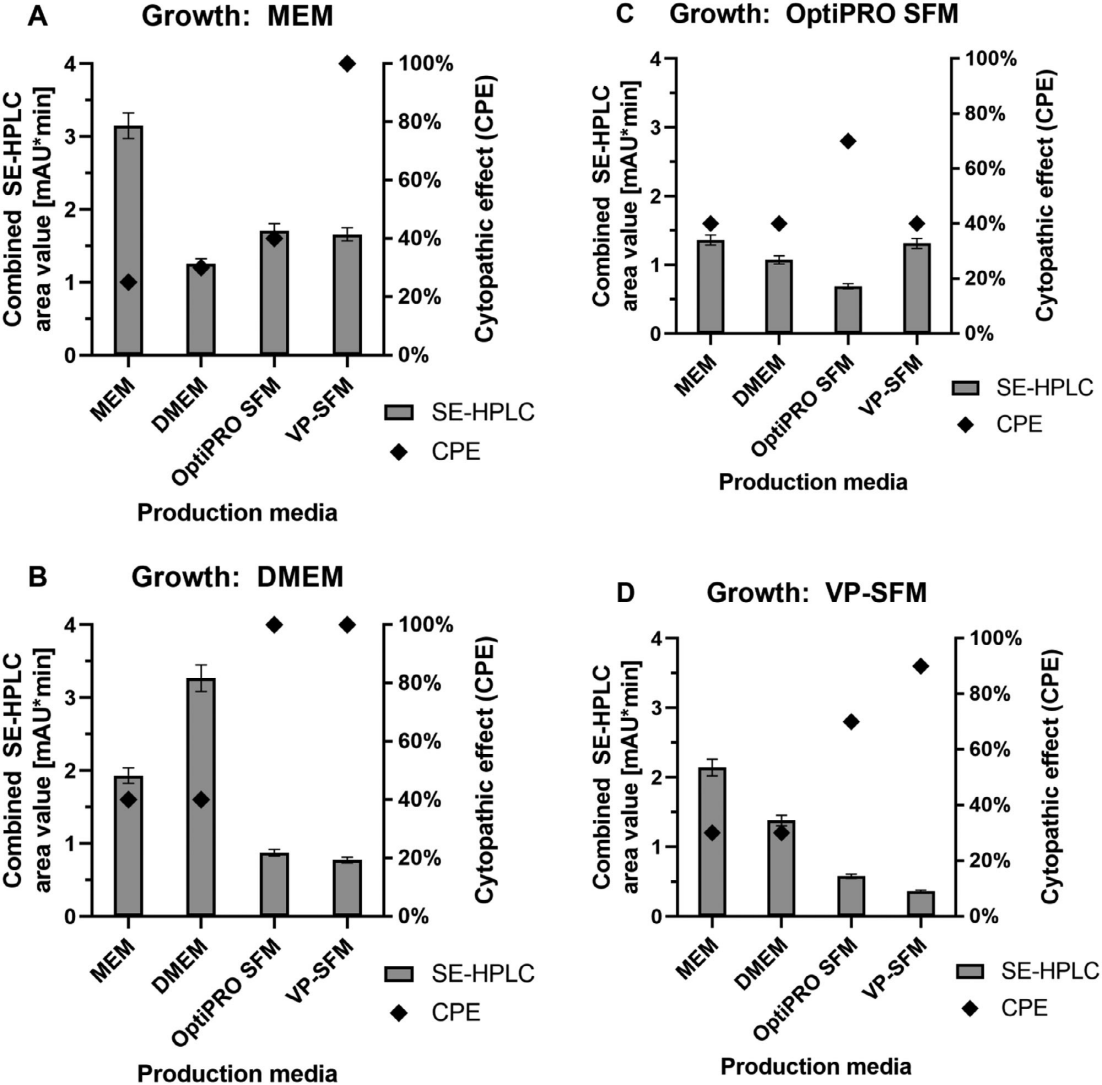
Four different growth media (A: MEM (+FBS); B: DMEM (+FBS); C: OptiPRO SFM; D: VP‐SFM) and production media (MEM, DMEM, OptiPRO SFM, VP‐SFM) tested as combinations in roller bottles. Determined pooled harvest (D3+D5+D7+D9) SE‐HPLC area values (grey bars), plus Day 9 estimated CPE data (black rhombus) are shown, with one‐fold standard deviation.

We observed post‐infection a high variability of cytopathic effect (CPE) estimations and yields of combined harvests (Day 3+5+7+9) qualified by SE‐HPLC (Figure 2). Day 9 CPE data ranged from 25 % to 100 % compared to cell numbers at infection. The highest SE‐HPLC area values were measured with a moderate CPE of 30–40 %. Media combinations with higher CPE values had lower yields, apart from MEM+VP‐SFM, which showed no visibly attached cells remaining (100 % CPE) at final Day 9. SFM used for production showed, on average, higher CPE values than MEM or DMEM (MEM: 34 %; DMEM: 35 %; OptiPRO SFM: 70 %; VP‐SFM: 83 %), which led, in general, to lower virus yields. For FBS‐supplemented media, two superior combinations were determined. MEM+MEM and DMEM+DMEM showed more than 3.0 mAU*min, whereas MEM or DMEM in combination with one of the SFM options resulted in lower area values of ∼1.7 mAU*min for MEM and ∼0.8 mAU*min for DMEM (Figure 2A,B). Using SFM as a growth medium resulted in low productivities in almost all combinations (Figure 2C,D). Only VP‐SFM+MEM showed 2.1 mAU*min as the third highest productivity overall compared to the other OptiPRO SFM or VP‐SFM combinations, with low values ranging from 0.4 to 1.4 mAU*min.

### 3.3 iCELLis Nano—Cell Growth

Preliminary cell expansion data revealed higher cell numbers for DMEM compared to MEM, as well as identical cell growth profiles with 5–10 % FBS supplementation (data not shown). Therefore, we chose to use DMEM with 5 % FBS as growth media in all serum‐containing iCELLis Nano experiments.

**FIGURE 3:**
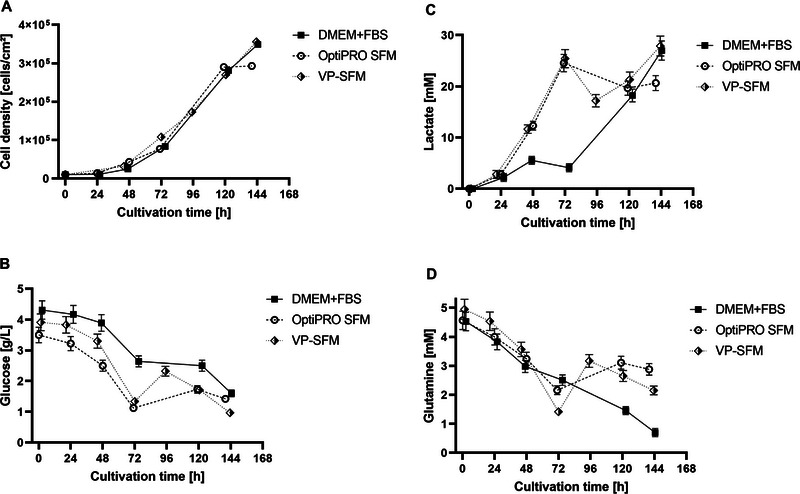
iCELLis Nano growth data using DMEM+FBS (black square, solid line); OptiPRO SFM (white circle, dashed line); and VP‐SFM (white and black rhombus, dotted line). (A) Cell growth kinetics. (B) Glucose kinetics. (C) Lactate kinetics. (D) Glutamine kinetics. One‐fold standard deviation is shown if not too low to visualize.

Cell growth and metabolite kinetics of DMEM(+FBS), OptiPRO SFM, and VP‐SFM culture are shown in Figure 3 and had a comparable progression. Doubling times were similar between DMEM(+FBS) and VP‐SFM culture at 28.1 and 27.8 h, respectively. Both growth curves peaked at Day 6 post‐inoculation, with a cell density around 3.5 × 10^5^ cells/cm^2^. Experiments with extended cultivation time (>6 days) resulted in a stationary phase with no further cell growth (data not shown). On Day 5, OptiPRO SFM had already reached its cell growth peak of 2.9 × 10^5^ cells/cm^2^, with no further cell growth on Day 6 but a doubling time of 28.6 h.

Total media volumes were appropriate, so that low metabolite concentrations (lower than 1 g/L glucose or 1 mM glutamine) were measured only just before infection. Time of infection was defined using cell density and metabolite data. More than 3.0 × 10^5^ cells/cm^2^ were targeted as high cell density production in combination with a lactate accumulation over 25 mM. For DMEM(+FBS) and VP‐SFM, lactate concentrations higher than 25 mM (DMEM: 27.0 mM; VP‐SFM: 27.9 mM) were reached on Day 6 just before infection.

### 3.4 iCELLis Nano—Virus Production

Considering the roller bottle data (Figure 2), we decided to use DMEM+DMEM and VP‐SFM+MEM as the best media combinations for iCELLis Nano virus production. However, because of low glucose (1.0 g/L) and nutrient content in MEM, we also assessed the combination of VP‐SFM+DMEM (4.5 g/L glucose) to compensate for possible nutrient limitations in view of high cell density production with more than 3.0 × 10^5^ cells/cm^2^.

**FIGURE 4:**
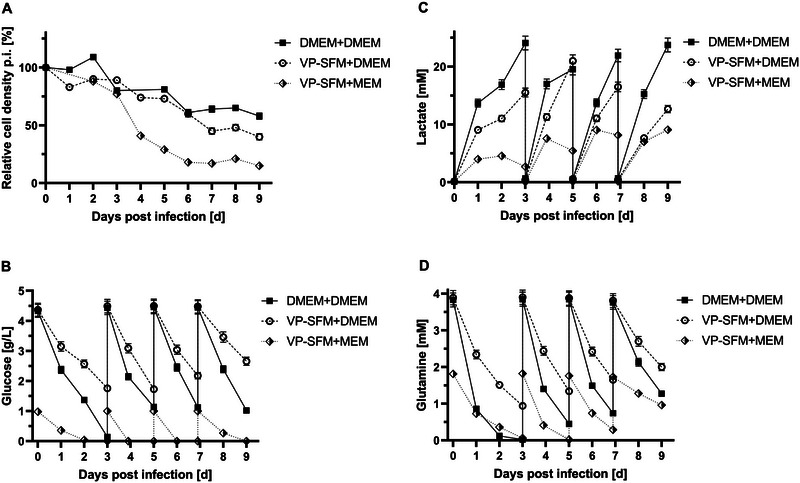
iCELLis Nano post infection (p.i.) data using DMEM+DMEM (black square, solid line); VP‐SFM+DMEM (white circle, dashed line); and VP‐SFM+MEM (white and black rhombus, dotted line). (A) Relative cell density kinetics post‐infection (cell count at infection was set to 100 %). (B) Glucose kinetics. (C) Lactate kinetics. (D) Glutamine kinetics. One‐fold standard deviation is shown if not too low to visualize.

Figure 4A shows the cell density course post‐infection. A reduction in cell numbers to 80 % for all three media combinations was seen until the first harvest on Day 3, before the measured cell counts progressed differently until the last harvest. On the final day, Day 9, the highest remaining cell count was measured for DMEM+DMEM, with 60 % cells remaining. With a steady decrease, 40 % of cells remained viable for VP‐SFM+DMEM as a media combination, but for VP‐SFM+MEM, < 20 % were counted on Day 9 because of a strong cell mass decrease after Day 3 post‐infection. Post‐infection metabolite data are shown in Figure 4B–D. Glucose levels were dropping to zero for DMEM+DMEM and VP‐SFM+MEM until Day 3 post‐infection. VP‐SFM+DMEM still had residual glucose present on Day 3 p.i. No glucose limitation was seen for this media combination in the following days after media exchanges. This applies as well for DMEM+DMEM after Day 3 p.i. for which no additional glucose limitation was detected. However, VP‐SFM+MEM continued to be limited in glucose as measured after each media exchange until Day 9 p.i. Maximum lactate levels between 15 and 25 mM were determined for the two DMEM media combinations, whereas MEM did not reach more than 10 mM. Highest glutamine consumption was noted by DMEM+DMEM with a limitation on Day 3 as seen for glucose levels before. VP‐SFM+DMEM did not show any limitations. VP‐SFM+MEM was limited in glutamine, but on fewer days compared to the glucose measurements.

**FIGURE 5:**
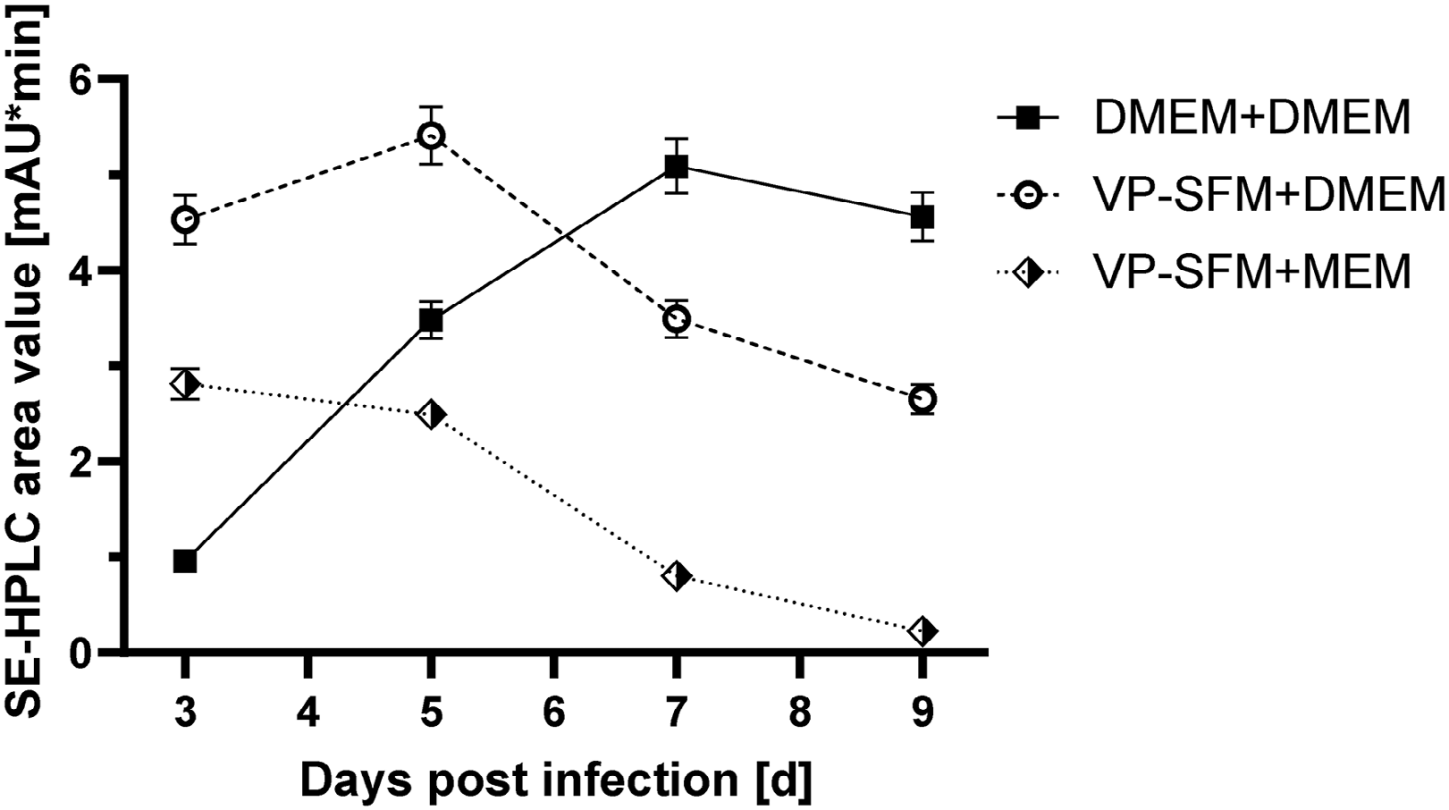
SE‐HPLC virus peak area comparison among DMEM+DMEM (black square, solid line); VP‐SFM+DMEM (white circle, dashed line); and VP‐SFM+MEM (white and black rhombus, dotted line) in iCELLis Nano, with one‐fold standard deviation. Harvest day values post‐infection are shown.

Determined virus peak area values showed different viral kinetics depending on the media used (Figure 5). For DMEM+DMEM, an intense increase in SE‐HPLC area was observed for each consecutive harvest. The peak was on Day 7, with 5.1 mAU*min. On Day 9, still 4.6 area units were measured. In contrast, VP‐SFM+DMEM reached a high value of 4.5 mAU*min on Day 3. The maximum value was measured on Day 5 at 5.4 mAU*min, decreasing to 3.5 area units on Day 7 and 2.7 mAU*min on Day 9. The maximum value for VP‐SFM+MEM was 2.8 area units on Day 3. On Day 5, a similar value of 2.5 mAU*min was determined before viral production declined to low area units on Days 7 and 9 (0.8 and 0.2 mAU*min, respectively).

**FIGURE 6:**
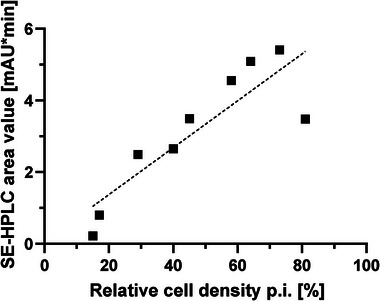
Relative cell density post infection (p.i.) versus SE‐HPLC virus peak area from three different media combinations (DMEM+DMEM, VP‐SFM+DMEM, and VP‐SFM+MEM) in the iCELLis Nano (black squares). Harvest day data (Days 5, 7, 9) were used and linear fitted (dotted line).

The relationship between cell numbers post‐infection (Figure 4) and SE‐HPLC virus peak area (Figure 5) is summarized in Figure 6. Correlational analysis showed a strong relationship between relative cell counts post‐infection and measured virus area (*R* ^2^ = 0.94). The higher the remaining cell density (or the weaker the cell lysis), the higher the measured viral productivity.

### 3.5 Productivity Evaluation Between the iCELLis Nano and Roller Bottles

**FIGURE 7:**
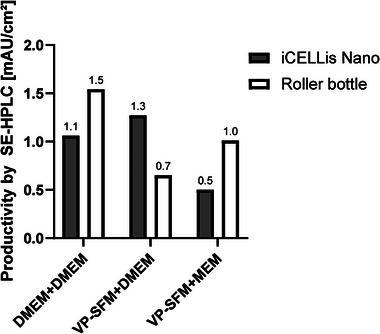
Viral productivity comparison between iCELLis Nano (grey bars) and roller bottles (white bars) in terms of SE‐HPLC area values per cm^2^. Data were evaluated from three different media combinations (DMEM+DMEM, VP‐SFM+DMEM, and VP‐SFM+MEM).

Productivity per surface area for both systems was highly dependent on the media combination used (Figure 7). DMEM+DMEM production resulted in 1.1 mAU/cm^2^ in the iCELLis Nano, whereas for roller bottles, there was about 40 % more yield per area, with 1.5 mAU/cm^2^ reaching the highest productivity overall. VP‐SFM culture with DMEM as the production media led to the highest serum‐free productivity with 1.3 mAU/cm^2^ in the iCELLis Nano compared to 0.7 mAU/cm^2^ with roller bottles. In contrast, productivity with VP‐SFM+MEM was 1.0 mAU/cm^2^ in roller bottles, but only 0.5 mAU/cm^2^ in the iCELLis Nano.

## 4 Discussion

### 4.1 SFM Establishment

Implementation of SFM in routine Vero cell culture required initial adaptation and optimization efforts. Direct adaption was performed for OptiPRO SFM and VP‐SFM, as previously suggested [17]. Optimization of the detachment method identified direct enzyme removal as the best strategy. This method could be applied to both media and plasticware tested (T‐flasks or roller bottles), regardless of the detachment solution used (TrypLE or Accutase). Rourou et al. [18] examined both enzymes as favorable SFM detachment solutions when using a short incubation time with subsequent removal. In that work, TrypLE was associated with higher average cell division numbers and thus has been used for routine cell culture. In our work, we calculated similar doubling times for both enzymes, but the higher aggregation levels seen with TrypLE led us to use Accutase for subsequent experiments. It must be noted that Accutase is of animal origin, which can be a safety and regulatory issue [11, 14]. As shown in this and other studies, TrypLE can be used instead as a recombinant alternative [4, 17].

### 4.2 Roller Bottle Media Screening

Sixteen media combinations were tested to determine productivity of JEV in roller bottles and decide on the final media for iCELLis Nano experiments. Results showed a high variability in CPE values and titers depending on the media combination used. Particularly, yields were lower with all SFM combinations apart from VP‐SFM+MEM. Homologous combinations of MEM+MEM or DMEM+DMEM with serum as a growth supplement resulted in the highest measured productivity. Heterologous usage of MEM or DMEM led to average productivity, which was unexpected as both media are similar in chemical composition and differ only in the higher nutrient concentrations and glycine and serine as extra amino acids for DMEM. These slight differences in media composition appeared to strongly affect viral yield, however. One explanation could be that cell adaptation processes are necessary for heterologous media combinations, which negatively influence virus production. Potential influential factors in viral productivity include the complexity of SFM (usually of undisclosed composition) and consequent unknown nutrient limitations, lot‐to‐lot differences, or changes in media osmolality [19]. In addition, at a low MOI (0.01), such as that used in this study, only a fraction of cells will be infected in the first infection cycle, leaving most cells in a growth metabolic state with unknown consequences if a media change interferes with metabolism [20, 21].

High variability in CPE data (25–100 %) gives further evidence that media choice can be crucial for virus production. Moderate CPE values (30–40 %) were beneficial for obtaining high titers, whereas stronger CPE levels led to lower yields, as seen for serum‐free production media compared to MEM or DMEM. Quesny et al. [22] also noted this increase in CPE rate for SFM in their study of the metabolic kinetics of Vero cells grown under serum‐free conditions. Particularly in that work, glutamine and serine were rapidly depleted with SFM in comparison to cells cultured with FBS. Both amino acids can be linked to cell apoptosis triggered by limitations in producing intermediate metabolic products [22]. As the composition of both tested SFM is undisclosed, only MEM and DMEM can be investigated. MEM does not include serine as an amino acid, whereas DMEM does. In our experiments, the absence of serine in MEM did not lead to higher cell death rates in comparison to DMEM (25–40 % CPE for both media). CPE levels of 100 % were observed only for media combinations with OptiPRO SFM or VP‐SFM, for which limiting media components such as serine cannot be ruled out.

Supplementary data generated in a similar experiment using another viral agent (data not shown) indicated another media combination pattern. FBS‐containing groups showed superior titers independently, if used in homologous or heterologous combinations. In contrast, SFM combinations resulted in rather low titers except with DMEM as production media, which showed high yields for both SFMs (OptiPRO+DMEM or VP‐SFM+DMEM) tested.

It becomes evident that the impact of media on viral productivity is more complex than originally expected and can vary for different viruses, so that individual media optimization is obligatory to improve yields. This inference has been confirmed by groups that have studied the choice of media and parameters affecting viral production for different host cells and viruses in terms of the so‐called cell‐density effect. This effect describes a lower specific viral productivity in high‐cell‐density experiments, which can be mitigated if a complete media exchange is performed before infection, as we did here by including an additional PBS wash [19, 21, 22]. Our data suggest that choice of media strongly affects viral yield, even if the media is exchanged properly before infection.

### 4.3 iCELLis Nano—Cell Growth and Production Phase

Implementation of an iCELLis Nano standard cell growth process showed similar results for all three media (DMEM, VP‐SFM, and OptiPRO SFM). Average doubling times were 27.5 h, and cell densities were 3.0 × 10^5^ cells/cm^2^ before infection, higher than previously reported cell densities for this system [24]. For OptiPRO SFM, an earlier stationary phase was noted (Day 5) in comparison to DMEM or VP‐SFM, for which cell growth kinetics were reproducibly stable, peaking at Day 6. Additional cell growth after Day 6 was not seen, probably because of lactate accumulation, which inhibits Vero cell growth at increasing concentrations [20].

At final harvest, VP‐SFM+DMEM yielded higher cell counts than VP‐SFM+MEM (40 % vs. 20 %) and higher yields, which can likely be explained by the higher nutrient content of DMEM. In roller bottles, 70 % of cells remained viable for both combinations until Day 9. This production system difference might trace to higher cell densities and a corresponding faster nutrient depletion in the iCELLis Nano system compared to roller bottles, for which DMEM appears better suited.

The analyzed metabolite data post‐infection shows that the low glucose concentration of MEM (1 g/L) is limiting, and even a high glucose DMEM formulation of 4.5 g/L as used in this study, can be insufficient in the first days post‐infection. A steady nutrient availability with no limitations, particularly in glucose or glutamine levels, is essential to maintain cells and generate high virus titers and could be further optimized by replacing media as soon as metabolite levels drop under a certain threshold. Additional mechanistic medium component analysis should be targeted by future work to elaborate in‐depth on which components influence cell metabolism and virus production for JEV. As pointed out by Shen et al. [23], nutrient consumption is complex and identifying limiting components can be challenging. Especially if the medium consists of undefined and complex supplements like hydrolysates. Another way of optimizing virus production could be by mixing different commercial media with each other and investigate if a media blend is superior in terms of productivity [25].

The remaining cells correlated with the determined virus peak area values over multiple harvests. The higher the cell numbers post‐infection, the higher the viral productivity measured as indicated by DMEM+DMEM production, with the highest remaining cell count of all three media tested and a strong virus production until final harvest.

Feasible production of JEV with high titers thus can be achieved when using DMEM (FBS‐supplemented) or VP‐SFM as growth media, as previously described for a different packed‐bed bioreactor [26]. Moreover, JEV can be added to the list of viruses that can be successfully produced in the iCELLis Nano [2].

### 4.4 Productivity Evaluation Between iCELLis Nano and Roller Bottles

Both systems proved to be excellent production systems for JEV if the correct media was used for the growth and production phase. In this study, we developed superior media combinations for both systems, using serum‐containing media or SFM. Especially, serum usage in roller bottles yielded around 40 % more virus per growth area than the iCELLis Nano, a surprising finding indicating that roller bottles remain competitive, despite being viewed as a rather simple, less finely controlled, and conventional technology. Determined serum‐free productivity when using the iCELLis Nano system was competitive in terms of yield with serum‐usage in roller bottles and vice versa. A static fixed‐bed bioreactor seems to be favored for the sensitive nature of cells cultured under serum‐free conditions, as it offers controlled setpoints for pH and dissolved oxygen compared to the dynamic and uncontrolled roller bottle system.

All productivity comparisons depended highly on the media combination used, whether roller bottles or the iCELLis Nano were used as the production system. Nevertheless, when considering large‐scale manufacturing, the preferred production system is a fixed‐bed bioreactor because of the lower footprint and greater scale. Fixed‐bed bioreactors up to 500 m^2^ are commercially available, whereas manufacturing in roller bottles is limited in scalability, achieving usually much smaller surface areas [8]. However, production in an iCELLis 500+ system needs to fit in the desired scale. No intermediate or pilot scale system is available between iCELLis Nano and iCELLis 500+, increasing the requirements to a proper downscale model for process characterization activities. Since consumable costs are relatively expensive for the iCELLis 500+, process characterization and scaling up in this system while performing engineering runs can result in quite an upfront investment. For clinical trial material manufacturing, another technology could be more feasible if another scale is needed [27]. Besides the dependency of a sole manufacturer for consumables like the single‐use unit, a big drawback for this kind of system is the inaccessibility of the cell culture itself. Assessment of cell distribution or limitations in nutrient diffusion throughout the fixed‐bed is not possible, resulting in a black box, although a biomass probe is available, which can give some additional information. For the iCELLis Nano macrocarriers can be collected from the top of the fixed‐bed to determine cell numbers. This is not possible in the closed, large‐scale system, which completely relies on offline metabolite measurements to assess the cell culture.

While knowing these limitations, the iCELLis system still can be a great production system with a low footprint while maintaining up to 500 m^2^ growth area due to its unique design, maintaining high cell densities with low shear stress levels [27]. As shown in this study, high virus yields are possible with this production system.

Finally, high‐cell‐density production needs to be evaluated for iCELLis Nano cultivation and coherent media selection. MEM cannot be recommended as media because of the low nutrient content that apparently limits virus production.

## 5 Concluding Remarks

Our work shows that Japanese encephalitis virus production reached increased productivity when using optimized medium combinations utilizing DMEM as the production medium. Its enriched glucose content beneficially affected viral yields, enabling potential large‐scale manufacturing using the fixed‐bed reactor with serum‐containing (DMEM+DMEM) or serum‐free media (VP‐SFM+DMEM). Thus, investigating the interplay of media composition, host cells, and viruses offers a better understanding of viral replication as a way to optimize productivity. In particular, with respect to completely SFM, an ideal production system must be found that provides suitable media combinations for individual virus production.

## Conflicts of Interest

The authors declare no conflicts of interest.

## Data Availability

The data that support the findings of this study are available from the corresponding author upon reasonable request.
